# ASO Author Reflections: Peritoneal Disease Control after Complete Cytoreductive Surgery in Patients with Colorectal Cancer and Extraperitoneal Metastases

**DOI:** 10.1245/s10434-026-19852-2

**Published:** 2026-05-25

**Authors:** M. Daniyal Tanweer, Mustafa Raoof

**Affiliations:** Department of Surgery, City of Hope National Medical Center, Duarte, CA USA

## Past

Cytoreductive surgery (CRS) for colorectal cancer peritoneal metastases (CRC-PM) is significantly associated with improved survival for peritoneal-limited disease.^1^ Complete macroscopic resection is strongly associated with enhanced disease control. Candidacy for CRS is evaluated through multidisciplinary tumor boards at expert centers, and generally requires adequate functional status, disease response or control with preoperative chemotherapy, and the technical feasibility of complete macroscopic resection with limited morbidity.

However, a major proportion of patients with peritoneal disease also have some form of extraperitoneal metastases (EPM), most commonly hepatic or pulmonary. Patients with concomitant extraperitoneal disease are associated with more aggressive disease and poorer survival outcomes. Therefore, historically, this patient population had been excluded from studies investigating the utility of CRS in disease control for CRC-PM, largely due to the understanding that EPM reflects disseminated systemic disease and as a result does not derive meaningful benefit from CRS.^2^

Advances in minimally invasive surgical techniques and systemic therapies have allowed surgeons to expand the indications for CRS. Combined peritoneal and liver resections are being increasingly performed for patients with multiorgan metastases, though recurrence rates remain high. However, the role of CRS for patients with co-existing EPM remains insufficiently defined. The objective of our study was to determine the efficacy of a complete CRS in providing locoregional disease control in patients with concomitant extraperitoneal disease and consequently help inform patient selection criteria.

## Present

Our single-institution, retrospective study included 83 patients undergoing curative intent CRS for CRC-PM.^3^ Patients were stratified by the presence of EPM at any point in their treatment history into two groups: peritoneal disease only (PDO) and co-existing EPM. Within the EPM cohort, liver-only metastases were most common (58%), followed by lung-only metastases (26%), with nodal and multisite involvement observed less frequently.

Primary outcomes were peritoneal progression-free survival (pPFS) and progression-free survival (PFS). Unadjusted analyses revealed comparable peritoneal disease control among the groups. Entropy balancing was utilized to further control for known prognostic confounders. This adjusted analysis also revealed that complete CRS conferred comparable pPFS for patients with and without EPM. Patients with EPM had shorter PFS, largely owing to extraperitoneal sites of progression. Expectedly, EPM was also associated with reduced overall survival on unadjusted and adjusted analyses.

On the basis of these findings, our study reports evidence that CRS can achieve effective locoregional control even in the setting of EPM. Because peritoneal disease affects quality of life and eligibility for future therapies, our study suggests that effective peritoneal disease control can be established in carefully selected patients given complete cytoreduction is achieved. However, patients should be counseled regarding the increased risk of extraperitoneal progression following CRS.

## Future

Our findings support the role of complete CRS for effective locoregional control in selected patients with EPM. Given the high rate of systemic progression in this cohort, swift return to systemic therapy is critical. Delay in return to systemic therapy has been highlighted as one of the main reasons for the lack of survival benefit of optimal or maximal tumor debulking in the ORCHESTRA trial.^4,5^ With continued advancements in minimally invasive surgical techniques for CRS (e.g., robotic CRS), more patients may be able to achieve meaningful disease control via complete CRS while ensuring fast recovery and return to systemic therapy. Prospective multi-institutional studies are needed to validate the findings of our study, understand the factors that influence the risk of extraperitoneal progression following CRS, and better define the quality-of-life implications of aggressive surgical intervention in patients with disease beyond the peritoneum.
